# Spatial cell interplay networks of regulatory T cells predict recurrence in patients with operable non-small cell lung cancer

**DOI:** 10.1007/s00262-024-03762-x

**Published:** 2024-08-02

**Authors:** Siqi Cai, Guanqun Yang, Mengyu Hu, Chaozhuo Li, Liying Yang, Wei Zhang, Jujie Sun, Fenghao Sun, Ligang Xing, Xiaorong Sun

**Affiliations:** 1 Shandong University Cancer Center, Shandong University, Jinan, Shandong China; 2 Shandong Cancer Hospital and Institute, Shandong First Medical University and Shandong Academy of Medical Sciences, Jinan, Shandong China; 3 School of Clinical Medicine, Weifang Medical University, Weifang, Shandong China; 4 Shandong Cancer Hospital and Institute and Shandong Academy of Medical Science, Jinan, Shandong China; 5 Department of Pathology, Shandong Cancer Hospital and Institute, Shandong First Medical University and Shandong Academy of Medical Science, Jinan, Shandong China; 6 Department of Radiation Oncology, Shandong Cancer Hospital and Institute, Shandong First Medical University, and Shandong Academy of Medical Sciences, Jinan, Shandong China; 7 Department of Nuclear Medicine, Shandong Cancer Hospital and Institute, Shandong First Medical University and Shandong Academy of Medical Sciences, No.440, Jiyan Road, Huaiyin District, Jinan, 250117 Shandong China

**Keywords:** Regulatory T cell, Cell spatial interplay network, Multiplex immunofluorescence, Tumor microenvironment, Lung cancer

## Abstract

**Background:**

The interplay between regulatory T cells (Tregs) and neighboring cells, which is pivotal for anti-tumor immunity and closely linked to patient prognosis, remains to be fully elucidated.

**Methods:**

Tissue microarrays of 261 operable NSCLC patients were stained by multiplex immunofluorescence (mIF) assay, and the interaction between Tregs and neighboring cells in the tumor microenvironment (TME) was evaluated. Employing various machine learning algorithms, we developed a spatial immune signature to predict the prognosis of NSCLC patients. Additionally, we explored the interplay between programmed death-1/programmed death ligand-1 (PD-1/PD-L1) interactions and their relationship with Tregs.

**Results:**

Survival analysis indicated that the interplay between Tregs and neighboring cells in the invasive margin (IM) and tumor center was associated with recurrence in NSCLC patients. We integrated the intersection of the three algorithms to identify four crucial spatial immune features [P_(CD8_^+^_Treg to CK)_ in IM, P_(CD8_^+^_Treg to CD4)_ in IM, N_(CD4_^+^_Treg to CK)_ in IM, N_(CD4_^+^_Tcon to CK)_ in IM] and employed these characteristics to establish SIS, an independent prognosticator of recurrence in NSCLC patients [HR = 2.34, 95% CI (1.53, 3.58), *P* < 0.001]. Furthermore, analysis of cell interactions demonstrated that a higher number of Tregs contributed to higher PD-L1^+^ cells surrounded by PD-1^+^ cells (*P* < 0.001) with shorter distances (*P* = 0.004).

**Conclusion:**

We dissected the cell interplay network within the TME, uncovering the spatial architecture and intricate interactions between Tregs and neighboring cells, along with their impact on the prognosis of NSCLC patients.

**Supplementary Information:**

The online version contains supplementary material available at 10.1007/s00262-024-03762-x.

## Introduction

T cells, functioning as immune supervisors and effectors, play a critical role in anti-tumor immunity by engaging in intricate interactions with other cells [1–3]. Through cytokines or direct contact with other cells within the tumor microenvironment (TME), T cells orchestrate the modulation of immune cell activities and lead to the death of tumor cells [4–6]. The interplay between T cells and other cells residing in the TME has been identified as a crucial point in the eradication of tumor cells, with mounting research indicating its intimate correlation with patient prognosis [7–9]. Therefore, mapping the architecture of T cells in the TME has greatly gained importance.

Regulatory T cells (Tregs) constitute an important T cell subset and exert a vital role in immune regulation, and their interaction with surrounding cells can significantly influence patient prognosis [10–13]. Recent studies have utilized cell-to-cell distance to investigate these interactions. For example, Nagl et al. found that shorter distances between Tregs and CD8^+^ T cells correlated with improved prognosis in patients with esophageal cancer [14]. Additionally, Feichtenbeiner et al. proposed that cell interactions occur within a specific range, as the proximity of Tregs to CD8^+^ T cells within a distance range of 30–110 μm was associated with favorable prognosis in gastric cancer patients [15]. However, in the context of non-small cell lung cancer (NSCLC), the leading cause of cancer-related death, an understanding of the reciprocal interactions between Tregs and surrounding cells, is scarce. Furthermore, Tregs are supposed to influence the efficacy of anti-PD-(L)1 immunotherapy covering all stages of NSCLC with unclear mechanisms [16–18]. Therefore, unraveling the intricate web of interactions between Tregs and neighboring cells contributes to exploring fresh avenues for precise therapeutics in NSCLC patients.

Eventually, in pursuit of a deeper comprehension of the latent biological mechanisms underlying the prognostic impacts of varied spatial arrangements, we delved into the intricate interplay between Tregs and neighboring cells and unveiled novel spatial parameters intricately linked to the recurrence of NSCLC. Subsequently, we formulated a predictive instrument, the spatial immune signature (SIS), to predict recurrence-free survival (RFS) across different stages, demonstrating robust and consistent performance. Additionally, in combination with clinical staging, the SIS held the potential for clinical applicability. Furthermore, we explored the correlation between PD-1/PD-L1 interaction and Tregs, aiming to offer novel insights into treatment strategies for NSCLC patients.

## Methods

### Samples and preparation of tissue microarrays (TMAs)

This study included patients with NSCLC who received radical surgery at the Shandong Cancer Hospital between January 2014 and October 2018. We implemented the eighth of the lung cancer classification for the identification of stages and the assessment of prognosis. The tissue samples of each patient who had not received neoadjuvant therapy were preserved using the formalin-fixed, paraffin-embedded (FFPE) method and only those with sufficient tissue remaining were used for TMA preparation. The two different cores of each TMA were chosen from two representative areas of the same patients containing the invasion margin (IM) and tumor center (TC). IM was delineated as the zone with a 1 mm radius, centered on the demarcation between malignant tissue and uninvolved tissue [19–21]. A total of 261 patients were ultimately included in the study excluding missing tissue and poor staining quality (Supplementary file 1: Fig. S1). Recurrence-free survival (RFS) was defined as the duration between the initial diagnosis and the first occurrence of recurrence or the conclusion of the follow-up period. This study was approved by the Ethics Committee of Shandong Cancer Hospital.

### Multiplex immunofluorescence staining and imaging processing

The expression intensity and spatial distribution of FOXP3, CD4, CD8, and CK were visualized by mIF staining in Panel 1, and FOXP3, PD-1, PD-L1, and CK were shown in Panel 2. The TMA blocks containing the IM and TC areas were sectioned into 3-μm-thick slices. TMA slides were melted, subjected to 12 h of dehydration at 60 °C, and then underwent deparaffinization with xylene and graded alcohol rehydration. Next, the slides were placed in heat-induced antigen retrieval using either ethylene diamine tetra acid buffer (pH 8.0) or citric acid buffer (pH 6.0) in a microwave oven. Antibody concentrations and staining sequences were pre-optimized for this experiment. Following continuous incubation with primary antibodies and horseradish peroxidase-conjugated secondary antibodies, tyramide signal amplification (TSA) was performed. After each round of TSA, slides were subjected to heat for antigen retrieval and antibody stripping. After all staining series, the multi-stained slides were counterstained with 4',6-diamidino-2-phenylindole (DAPI) and scanned via the Vectra Polaris scanning system. Two expert pathologists, who were unaware of the patient information, evaluated all NSCLC samples and excluded inappropriate staining.

The stained slides were scanned by Vectra Polaris Scanner System, and the subsequent multispectral images were processed using Inform software 2.4.8. The inform software constructed a spectral library using single-stained slides for each tissue fluorescence spectrum and extracted the phenotyping algorithm from all spectrally unmixed images. Subsequently, the acquired images were recognized tumor or stroma compartments based on pan-CK labeled epithelial cell markers and analyzed cell segmentation according to the combination of the nuclear signals and cell morphology features. Detailed information about all primary antibodies and equipment used are provided in Supplementary file 1: Tables S1 and S2

### Establishment of spatial immune characteristic parameters

We utilized mIF images and automated imaging processing system to identify various cell types and quantitatively analyzed cell density and spatial distribution within the TME (Supplementary file 2). Assuming A represented a specific cell type and B denoted another cell type, D_(A)_ represented the density of A cells and D_(B)_ represented the density of B cells in tissue site. Immune feature parameters of the mean nearest neighbor distance (mNND) and proximity score were established to explain the cell–cell interaction in the TME deciphered by the R software (version 3.6.3) phenoptrReports package, which was defined as follows:

$$N_{{\left( {A to B} \right)}} = {{\mathop \sum \limits_{th = n} d_{\min } \left( {A_{th} - B} \right)} \mathord{\left/ {\vphantom {{\mathop \sum \limits_{th = n} d_{\min } \left( {A_{th} - B} \right)} {n\left( A \right)}}} \right. \kern-0pt} {n\left( A \right)}}$$

The variable n signified the total cell count. The mNND calculated the average distance between all A cells and their closest neighboring B cells. Additionally, the expression ${d}_{min}\left({A}_{th}-B\right)$ referred to the minimum distance from each A cell to other B cells. A smaller value of ${N}_{(A to B)}$ indicated a stronger interaction between these cell types.

$$P_{{\left( {A {\text{to}} B} \right)}} = {{\mathop \sum \limits_{th = N} n\left( {C_{th} \xrightarrow{r} A} \right)} \mathord{\left/ {\vphantom {{\mathop \sum \limits_{th = N} n\left( {C_{th} \to ^{r} A} \right)} {N\left( B \right)}}} \right. \kern-0pt} {N\left( B \right)}}$$

The proximity score was computed as the mean count of A cells within a specific radius surrounding all B cells throughout the tissue site. Additionally, N denoted the total cell count, and r indicated the specified radius. n(C_th_ $\stackrel{r}{\to }$ A) represented the quantity of A cells within the r-um radius encircling each B cell. Therefore, an elevated proximity score for A cells signified an increased B cell density close to A cells within a defined distance, implying enhanced intratumoral infiltration.

### Selection of crucial features and construction of a spatial immune signature

To obtain a more refined and stable model, the cohort of 261 NSCLC patients was randomly classified into training (*N* = 183) and calibration (*N* = 78) groups, maintaining a ratio of 7:3. Crucially, we meticulously maintained the equilibrium of clinical characteristics within the training and validation cohorts (Supplementary Table S3). In the training cohort, we adapted three types of machine learning algorithms, including least absolute shrinkage and selection operator (LASSO) regression, extreme gradient boosting (XGBoost), and random forest (RF), to select the most important relevant immune features in R (version 3.6.3). Afterward, the intersecting immune parameters among the LASSO, XGBoost, and RF were considered the most critical immune characteristics and were visualized. Then, the intersection-based importance outcomes and multivariate Cox regression coefficients were combined to construct the spatial immune signature (SIS). The final formula was calculated as follows: SIS = 1.25 × [P_(CD8_^+^_Treg-CD4)_ in IM] + 0.70 × [P_(CD8_^+^_Treg-CK)_ in IM] – 0.67 × [N_(CD4_^+^_Treg-CK)_ in IM] – 0.56 × [N_(CD4_^+^_Tcon-CK)_ in IM]. The performance of the SIS was evaluated using receiver-operating characteristic (ROC) curves and the associated areas under the ROC curve (AUCs).

### Statistical analysis

Categorical data differences were assessed using the chi-square test and Fisher’s exact test. Continuous variable comparisons between two groups were conducted via the Mann–Whitney U test. Differences in RFS between the two groups were estimated using the log-rank test and the Kaplan–Meier method. The ROC and AUCs were implemented to evaluate the classification performance in the RFS of the model. The optimal cut-off value was determined using X-tile software (version 3.6.1). Data analysis and visualization were performed using SPSS (version 26.0), R (version 3.6.3), and GraphPad Prism (version 8.0). Statistical tests were two-sided, and a *P* value < 0.05 was considered significant.

## Results

### Patients clinicopathological characteristics

A total of 261 patients with stage I-III NSCLC who were recruited met the inclusion criteria among whom 103 (39.5%) cases relapsed during the follow-up time. The recurrence rates at 1, 3, and 5 years were 14.6%, 42.1%, and 62.5%, respectively. The majority of the cases were comprised of male (64.4%), lung adenocarcinoma (65.9%), and stage I (57.5%) patients among 261 patients with NSCLC, 165 (63.2%) patients received adjuvant chemotherapy, and 37 (14.2%) patients underwent adjuvant radiotherapy (Table 1).

**Table 1:** Clinicopathological characteristics of 261 patients with samples subjected to multiplex staining

Parameter	All patients (*N* = 261)
Age, y	
≤ 65	177 (67.8%)
> 65	84 (32.2%)
Gender	
Male	168 (64.4%)
Female	93 (35.6%)
Smoking index ^a^	
< 400	148 (56.7%)
≥ 400	113 (43.3%)
ECOG PS	
0–1	72 (27.6%)
> 1	189 (72.4%)
Histological type	
LUSC	89 (34.1%)
LUAD	172 (65.9%)
T stage	
T1	87 (33.3%)
T2	149 (57.1%)
T3	13 (5.0%)
T4	12 (4.6%)
N stage	
N0	183 (70.1%)
N1	44 (16.9%)
N2	34 (13.0%)
N3	0 (0%)
AJCC stage	
I	150 (57.5%)
II	63 (24.1%)
III	48 (18.4%)
Adjuvant chemotherapy	
Yes	165 (63.2%)
No	96 (36.8%)
Adjuvant radiotherapy	
Yes	37 (14.2%)
No	224 (85.8%)

*LUSC*, Lung squamous cell carcinoma; *LUAD*, lung adenocarcinoma

a Smoking index = number of cigarettes smoked per day × year(s)

### Overview of the distribution of Tregs in the tumor microenvironment

**Fig. 1:**
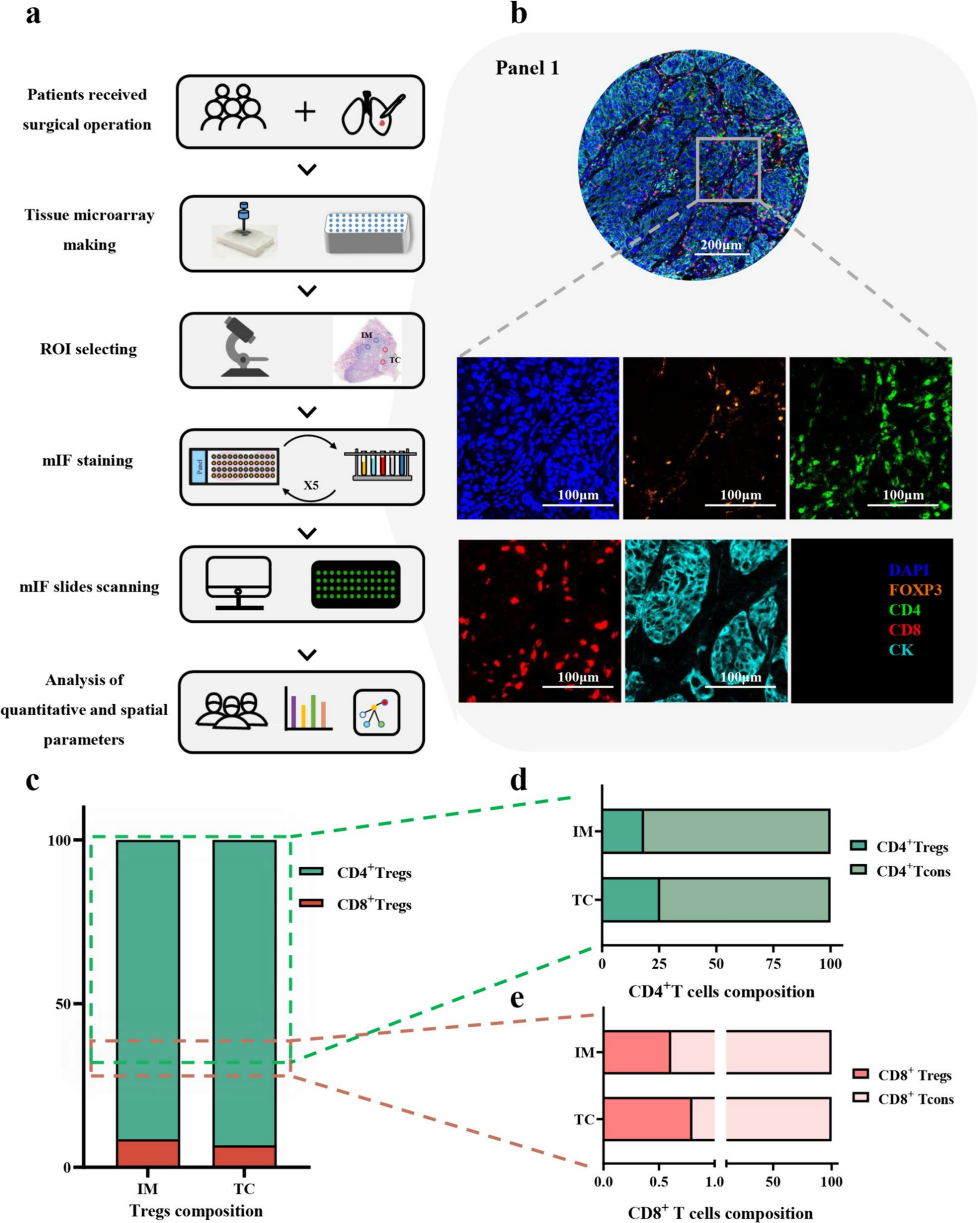
Characterization of tumor microenvironment in non-small cell lung cancer. **a** Study follow chart. ROI, region of interest (ROI). **b** Representative composite and single-stained images of Panel 1. **c-e** Relative distribution analysis of Tregs subsets in IM and TC. **d** CD4^+^Tregs. **e** CD8^+^Tregs

mIF staining was used to visualize the real immune landscape, and inForm software was used to digitize the spatial features of diverse cells in whole TMA sections (Fig. 1a). We analyzed the composition of various cells in panel 1 (Fig. 1b) and identified Tregs based on the specific marker FOXP3. The results showed that Tregs were predominantly composed of CD4^+^Tregs (IM, 91.4%; TC, 93.3%) and CD8^+^Tregs (IM, 8.6%; TC, 6.7%) and a considerable proportion of CD4^+^T cells and CD8^+^T cells expressed FOXP3 in IM and TC (Fig. 1c, e). Then, we employed the survival analysis to explore the relationship between CD4^+^Tregs or CD8^+^Tregs and recurrence. We found the D_(CD4_^+^_Tregs)_ in TC (65.03 vs. not reached, *P* = 0.011) and D_(CD8_^+^_Tregs)_ in IM and TC (IM, 68.00 vs. not reached, *P* = 0.018; TC, 62.03 vs. not reached, *P* = 0.001) were associated with the better prognosis of NSCLC patients via the log-rank test and the Kaplan–Meier method (Supplementary file 1: Fig. S2). The results from the univariate Cox analysis are similar to the aforementioned findings (Supplementary file 1: Table S4). Of note, in multivariate Cox regression, the interaction of PD-L1/PD-1 appears no significant relationship with the recurrence of NSCLC after adjusting for clinicopathological factors (including age, gender, histological type, smoking index and stage).

### Interaction between Tregs and neighboring cells was associated with the RFS of patients with NSCLC

**Fig. 2:**
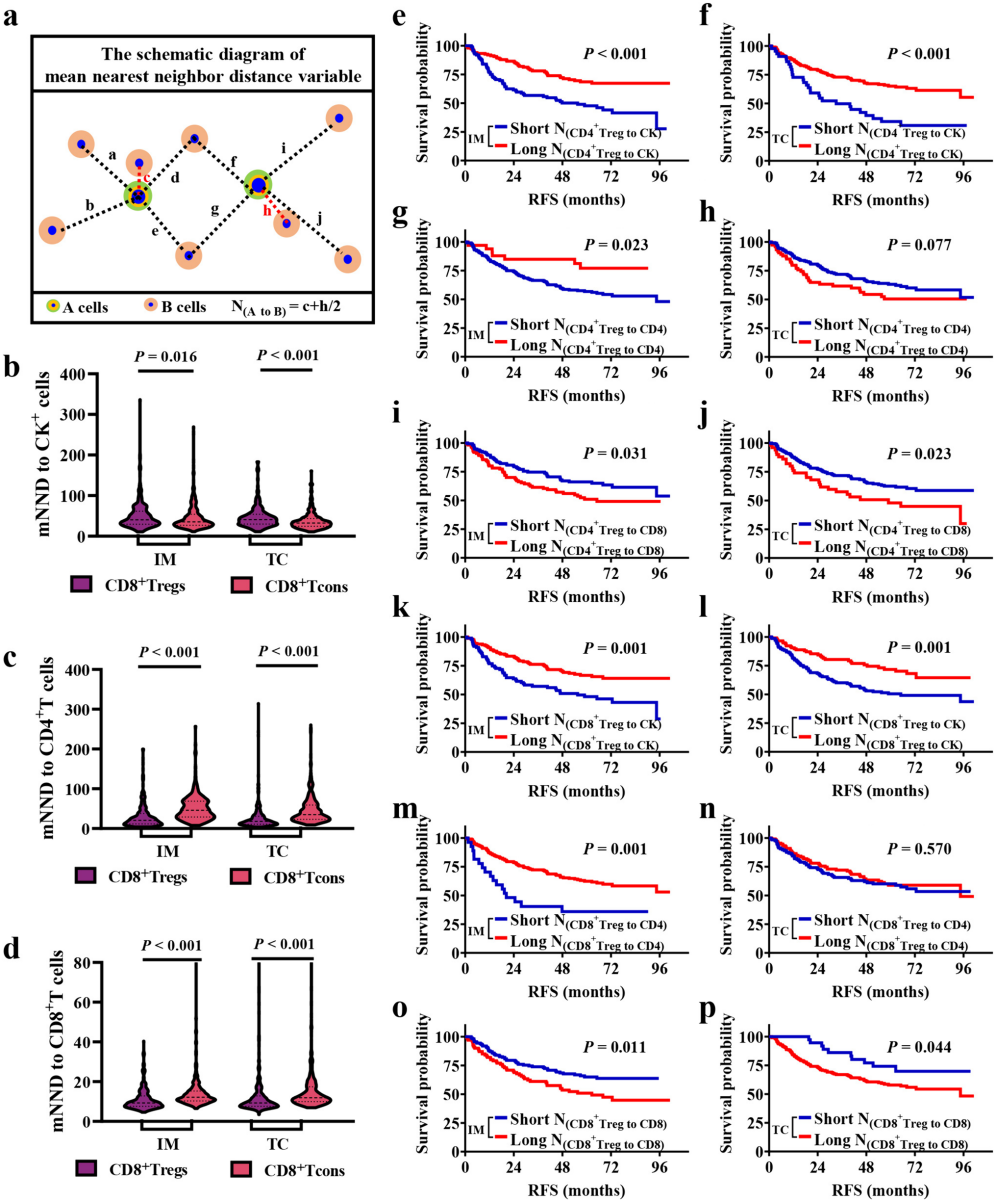
The association of mNND between Tregs and neighboring cells with RFS in NSCLC patients. **a** Illustration of the mNND involving Tregs and neighboring cells. **b–d** The discrepancy of the mNND from CD8^+^Tregs and CD8^+^Tcons to neighboring cells in IM and TC. **e–p** Kaplan–Meier curves of RFS based on the mNND of Tregs to neighboring cells. Comparison statistical analysis was determined by the Mann–Whitney U test, and cumulative RFS were calculated by log-rank test

Firstly, we developed the mNND parameter to quantify the strength of cell–cell interactions, with a shorter mNND indicating a more robust interaction between cells (Fig. 2a). Patients with more advanced clinical stages showed shorter distances between CD4^+^Tregs and tumor cells in IM and TC, and CD4^+^T cells in IM, while the distance between CD4^+^Tregs and CD8^+^T cells in IM and TC was prolonged. The distance between CD8^+^Tregs and tumor cells or CD4^+^T cells in IM and TC decreased with the advancing TNM staging, while the distance between CD8^+^Tregs and CD8^+^T cells in IM and TC increased (all *P* < 0.05; Supplementary file 1: Table S5). In addition, we observed no significant difference in the interaction between CD4^+^Tregs and tumor cells or T cells compared with CD4^+^T conventional cells (Tcons) in TME (Supplementary file 1: Fig. S3). Additionally, CD8^+^Tregs were observed to be more distant from tumor cells, while being closer to CD4^+^T cells and CD8^+^T cells in IM and TC (Fig. 2b–d). Moreover, the distance between CD4^+^Tregs (*P* < 0.001) or CD8^+^Tregs (*P* = 0.027) to tumor cells was longer in IM than in TC (Supplementary file 1: Fig. S4). Subsequently, we examined the impact of interactions between Tregs and surrounding cells on RFS in patients with NSCLC (Fig. 2e–p). The results demonstrated that shorter distances between CD4^+^Tregs and tumor cells in IM and TC (IM, 55.50 vs. not reached, *P* < 0.001; TC, 33.10 vs. not reached, *P* < 0.001), or between CD4^+^Tregs and CD4^+^ T cells in IM (94.47 vs. not reached, *P* = 0.023), were associated with poorer patient outcomes, which were in accordance with the mNND from the CD8^+^Treg to tumor cells in IM and TC (IM, 55.50 vs. not reached, *P* = 0.001; TC, 65.03 vs. not reached, *P* = 0.001), and to CD4^+^T cells in IM (20.30 vs. not reached, *P* = 0.001). Conversely, a shorter distance between CD4^+^Tregs (IM, not reached vs. 65.03, *P* = 0.031; TC, not reached vs. 58.90, *P* = 0.023) or CD8^+^Tregs (IM, not reached vs. 62.50, *P* = 0.011; TC, not reached vs. 94.47, *P* = 0.044) and CD8^+^T cells in IM and TC were correlated with longer RFS.

**Fig. 3:**
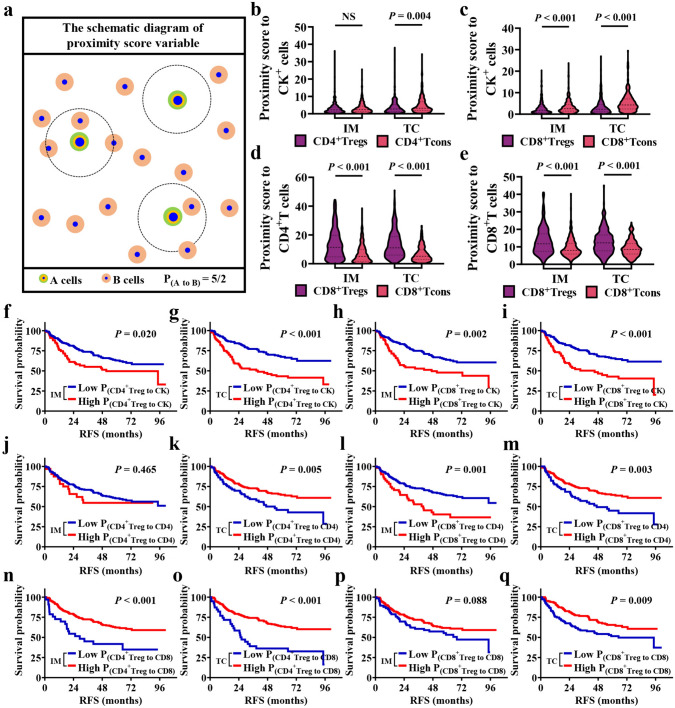
The association of proximity between Tregs and neighboring cells with RFS in NSCLC patients. **a** Illustration of the proximity score involving Tregs and neighboring cells. **b–e** The discrepancy of the proximity score from Tregs and Tcons to neighboring cells in IM and TC. **f–q** Kaplan–Meier curves of RFS based on the proximity score of Tregs to neighboring cells. Comparison statistical analysis was determined by the Mann–Whitney U test and cumulative RFS was calculated by log-rank test. NS, no significance

Secondly, the proximity score was also applied to reflect the interactions between cells. A higher proximity score indicates that the core cell is surrounded by more neighboring cells, implying stronger interactions between the central cell and its surroundings (Fig. 3a). With the TNM staging, CD4^+^Tregs or CD8^+^Tregs were surrounded with more tumor cells in IM and TC and CD4^+^T cells in IM. In contrast, CD4^+^Tregs surrounded with more CD8^+^T cells in IM and TC and CD8^+^Tregs surrounded with more CD8^+^T cells in TC (all *P* < 0.05; Supplementary file 1: Table S6). We compared the spatial characteristics between Tregs and Tcons and the results showed that CD4^+^Tregs in TC and CD8^+^Tregs in both IM and TC were surrounded by more tumor cells (Fig. 3b, c). Moreover, CD8^+^Tregs had more CD4^+^T cells and CD8^+^T cells in IM and TC (Fig. 3d, e). Interestingly, the proximity score of CD4^+^Tregs to tumor cells, CD4^+^T cells, and CD8^+^T cells were similar to CD4^+^Tcons (Supplementary file 1: Fig. S5). And there were more tumor cells surrounded CD4^+^Tregs (*P* = 0.005) and CD8^+^Tregs (*P* = 0.007) in TC than in IM (Supplementary file 1: Fig. S6). Similarly, we analyzed the relationship between the proximity score of Tregs to surrounding cells and patient prognosis. The survival analysis suggested that a higher number of tumor cells around CD4^+^Tregs (IM, 51.33 vs. no reached, *P* = 0.020; TC, 39.93 vs. not reached, *P* < 0.001) and CD8^+^Tregs (IM, 46.93 vs. not reached, *P* = 0.002; TC, 45.03 vs. not reached, *P* < 0.001) showed a lower RFS both in IM and TC (Fig. 3f–i). In contrast, increased CD4^+^T cells around CD4^+^Tregs (not reached vs. 54.03, *P* = 0.005) and CD8^+^Tregs (not reached vs. 46.80, *P* = 0.003) in TC were associated with better patient prognosis. Interestingly, in IM, patients with a higher amount of CD4^+^T cells around CD8^+^Tregs had a statistical correlation with worse RFS (39.93 vs. not reached, *P* = 0.001; Fig. 3j–m). Additionally, a higher number of CD8^+^T cells around CD4^+^Tregs in both IM and TC (IM, not reached vs. 29.93, *P* < 0.001; TC, not reached vs. 26.23, *P* < 0.001), as well as around CD8^+^Tregs in TC (not reached vs. 65.03, *P* = 0.009), were all correlated with longer RFS in patients (Fig. 3n–q).

### Tregs were correlated with the interplay between PD-1/PD-L1

**Fig. 4:**
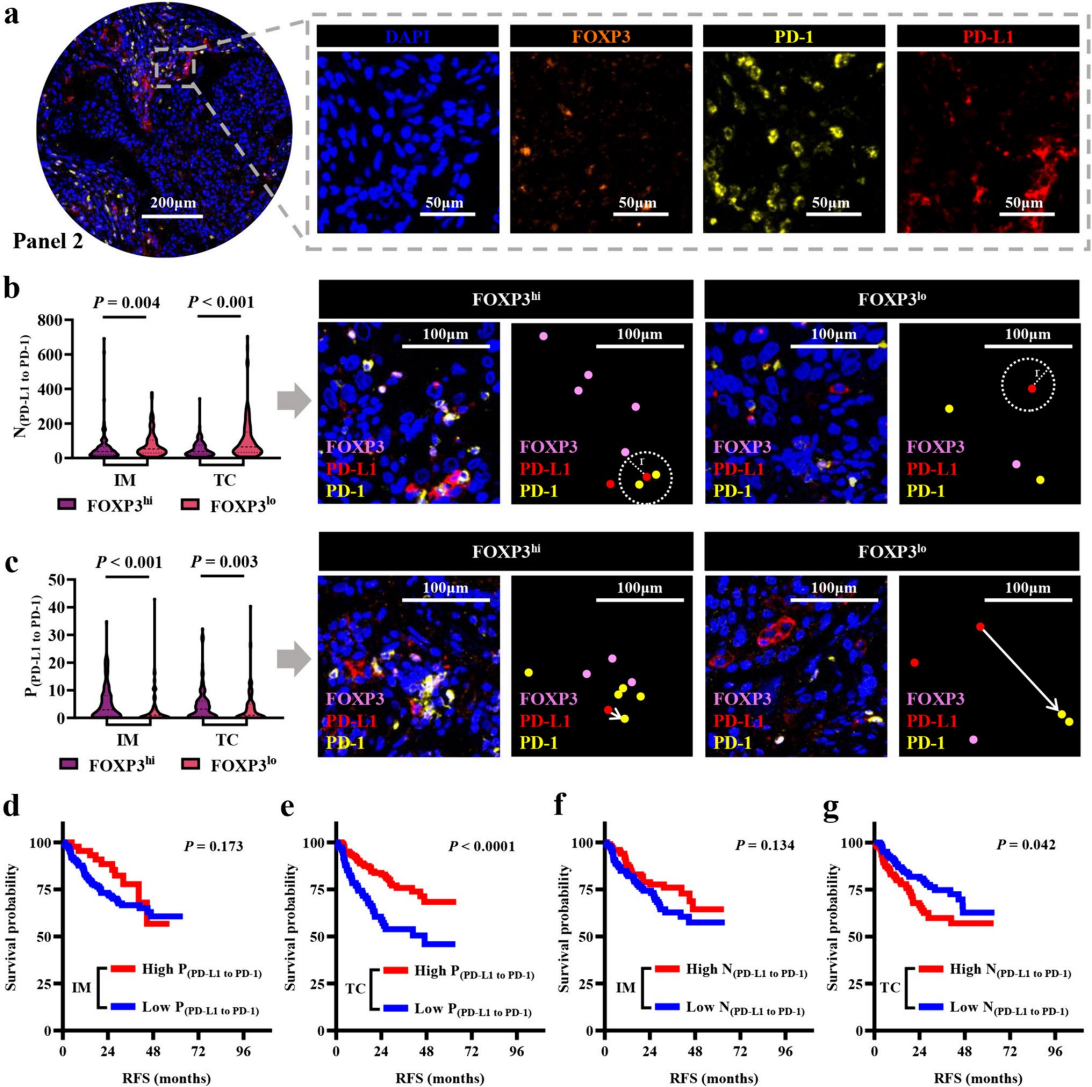
Discrepancy of the interplay of PD-1/PD-L1 between FOXP3 high expression group and FOXP3 low expression group. **a** Representative composite and single-stained images of Panel 2. **b** mNND from PD-L1^+^ cells to PD-1^+^ cells in IM and TC. **c** Proximity score from PD-L1^+^ cells to PD-1^+^ cells in IM and TC. **d**–**g** Kaplan–Meier curves of RFS based on the interaction of PD-L1/PD-1. r equated 30 μm. Significance (*P* value) was determined by the Mann–Whitney U test

Our above findings revealed a close association between Tregs and tumor cells concerning the prognosis of NSCLC patients and current research unveiled the interaction of PD-1/PD-L1 influencing the proliferation and function of FOXP3^+^ Treg cells [22–25], which could offer novel insights to enhance the efficacy of anti-PD-1/PD-L1 antibody treatments. Using another mIF panel (Fig. 4a), our investigation revealed a direct relationship wherein an elevated quantity of FOXP3^+^ cells corresponded to an increased abundance of PD-1 + cells and PD-L1 + cells (Supplementary file 1: Fig. S7). Notably, we compared the differences in spatial interactions between PD-1/PD-L1 within the high FOXP3 + cells group and the low FOXP3^+^ cells group. Our results manifested that high FOXP3^+^ cells contributed higher proximity score of PD-L1 + cells to PD-1 + cells (*P* < 0.001) and shorter mNND of PD-L1 + cells to PD-1 + cells (*P* = 0.004), which reflects the strong interplay between the PD-1/PD-L1 (Fig. 4b, c). We examined the impact of interactions between PD-L1 and PD-1 cells on RFS in patients with NSCLC. The Kaplan–Meier analysis showed that the low N_(PD-L1 to PD-1)_ in TC and high P_(PD-L1 to PD-1)_ in TC (46.93 vs. not reached, *P* < 0.001) were associated with favorable prognosis in patients with NSCLC (Fig. 4d–g). By univariate Cox regression, we found the high P_(PD-L1 to PD-1)_ in TC predicted longer RFS [HR = 0.68 (0.46, 0.99), *P* = 0.043, Supplementary file 1: Table S7].

### Spatial immune signature (SIS) contributed to predicting recurrence in patients with NSCLC

**Fig. 5:**
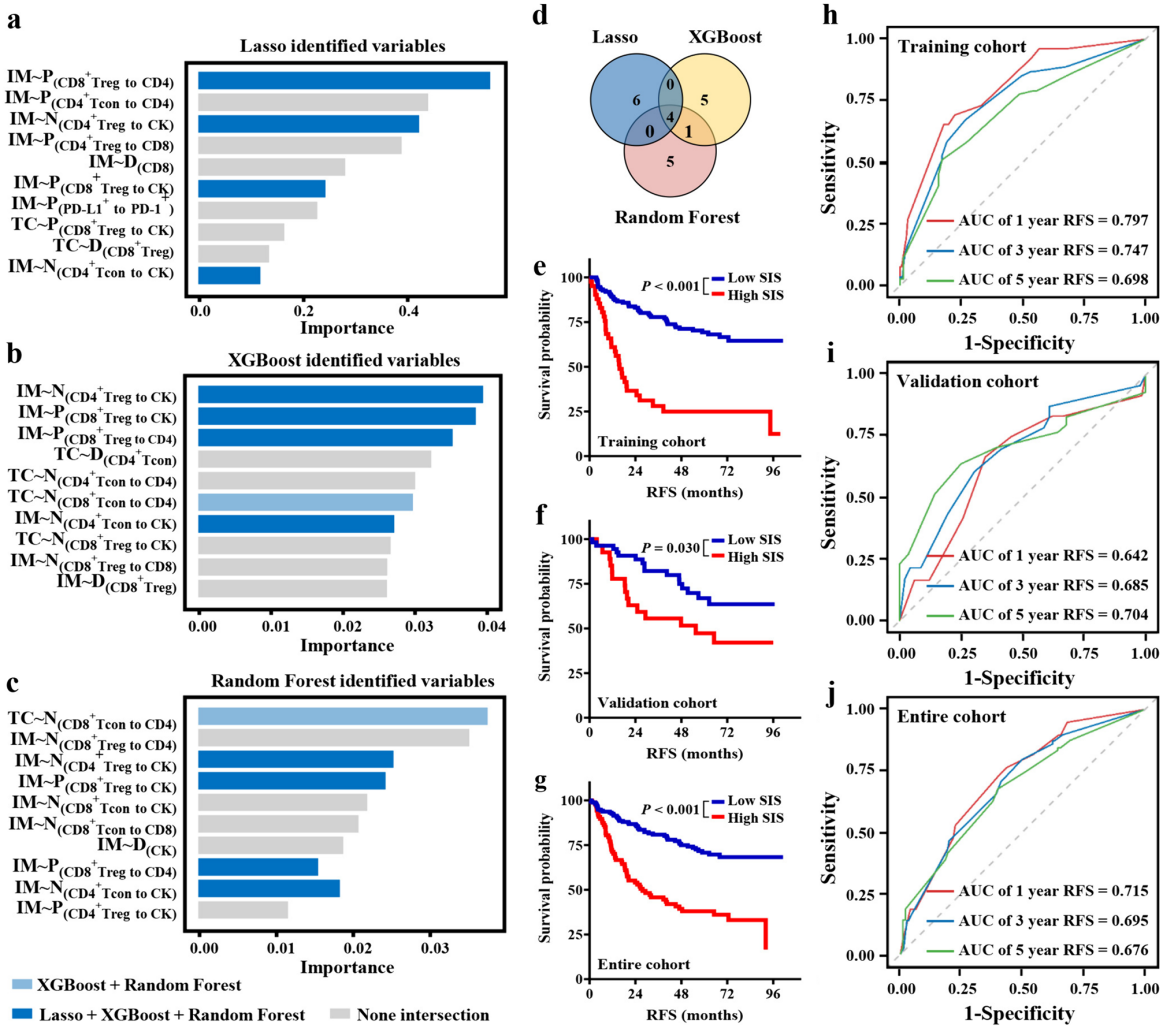
Construction and validation of the spatial immune significance (SIS). **a**–**c** The important feature map was selected by three machine learning algorithms (Lasso, XGBoost and RF). **d** Venn diagram identified the four most critical spatial immune features. **e**–**f** ROC and AUCs of SIS for prediction of recurrence risk at 1, 3, and 5 years in the training (**e**), validation (**f)**, and entire cohort (**g**). **h**–**j** Kaplan–Meier curves of RFS based on different SIS groups in the training (**h**), validation (**i**), and entire cohort. (**j**). Significance (*P* value) was determined using a Log-rank test

To comprehensively ascertain the effects of immune indicators on prognosis, we constructed spatial immune signature by three machine learning algorithms, including LASSO, XGBoost, and RF (Fig. 5a–c). We incorporated parameters including cell density and interaction of Tregs into the feature selection process of the model (Supplementary file 1:Table S8). The intersection of ten optimal variables with absolute values of nonzero coefficients selected by LASSO, and the top ten importance features extracted, respectively, by XGBoost and RF identified four critical immune parameters [P_(CD8_^+^_Treg to CD4)_ in IM, P_(CD8_^+^_Treg to CK)_ in IM, N_(CD4_^+^_Treg to CK)_ in IM and N_(CD4_^+^_Tcon to CK)_ in IM], which were applied to model construction (Fig. 5d). Univariate Cox regression manifested the higher P_(CD8_^+^_Treg to CD4)_ in IM [HR = 1.87, 95%CI (1.25, 2.78), *P* = 0.002], P_(CD8_^+^_Treg-CK)_ in IM [HR = 1.99, 95% CI (1.29, 3.06), *P* = 0.002] and shorter N_(CD4_^+^_Treg-CK)_ in IM [HR = 0.45, 95% CI (0.30, 0.66), *P* < 0.001] and N_(CD4_^+^_Tcon-CK)_ in IM [HR = 0.40, 95% CI (0.26, 0.63), *P* < 0.001] were associated with worse outcomes (Table 2).

**Table 2:** Univariate Cox regression demonstrated the prognostic effects of the critical spatial immune parameters

Variable	Univariate	
	HR (95%CI)	*P*
P_(CD8_^+^_Treg to CD4)_ in IM	1.87 (1.25, 2.78)	0.002
P_(CD8_^+^_Treg to CK)_ in IM	1.99 (1.29, 3.06)	0.002
N_(CD4_^+^_Treg to CK)_ in IM	0.45 (0.30, 0.66)	< 0.001
N_(CD4_^+^_Tcon to CK)_ in IM	0.40 (0.26, 0.63)	< 0.001

Then, the cases were split into a high-SIS group and a low-SIS group. Survival analysis showed that in the training (15.70 vs. not reached, *P* < 0.001), validation (55.50 vs. not reached, *P* = 0.003), and entire cohorts (27.70 vs. not reached, *P* < 0.001), the higher SIS group had a shorter RFS than the low-SIS group, respectively (Fig. 5e, f). Moreover, a multivariate Cox regression model of RFS was built based on NSCLC staging and SIS. Both NSCLC staging [HR = 2.26, 95% CI (1.51, 3.39), *P* < 0.001] and SIS [HR = 2.34, 95% CI (1.53, 3.58), *P* < 0.001] were significantly associated with RFS in NSCLC (Table 3). Additionally, we estimated its effectiveness by time-dependent ROC analysis. In the training set, the AUC values for 1-, 3-, and 5-year RFS were 0.797, 0.747, and 0.698, respectively, while in the validation set, they were 0.642, 0.685, and 0.704, respectively (Fig. 5h, i). Additionally, in terms of the entire dataset, the AUCs for predicting recurrence at 1, 3, and 5 years were 0.715, 0.695, and 0.676, respectively (Fig. 5j). Notably, the SIS system exhibited superior predictive capability compared to TNM staging and single indicators (Supplementary file 1: Figs. S8, S9). Collectively, the SIS consistently demonstrated stable and promising performance in distinguishing between NSCLC patients who experienced recurrence and those who remained recurrence-free after undergoing radical resection.

**Table 3:** Univariate and multivariate Cox regression models demonstrated the prognostic effects of the spatial immune signature

Variable	Univariate		Multivariate	
	HR (95%CI)	*P*	HR (95%CI)	*P*
Age				
< 65 years	Ref			
> 65 years	0.76 (0.50, 1.16)	0.203		
Gender				
Male	Ref			
Female	0.79 (0.53, 1.18)	0.244		
Histological type				
LUAD	Ref			
LUSC	1.17 (0.78, 1.73)	0.451		
Smoking index				
< 400	Ref			
≥ 400	1.09 (0.74, 1.59)	0.663		
Stage				
I-II	Ref			
III	2.67 (1.81, 3.96)	< 0.001	2.26 (1.51, 3.39)	< 0.001
SIS				
Low group	Ref			
High group	2.84 (1.88, 4.29)	< 0.001	2.34 (1.53, 3.58)	< 0.001

## Discussion

Through the application of mIF in combination with spatial assays driven by artificial intelligence, we identified a spectrum of cell phenotypes and explored spatial interaction patterns within the TME across a cohort of 261 NSCLC cases. Furthermore, we established an SIS to predict the prognosis of NSCLC patients. Additionally, we utilized an alternative staining panel to investigate the impact of Tregs on the interaction between PD-1/PD-L1. Our findings provided valuable insights into the spatial architecture and diversity of Tregs within the TME of NSCLC, emphasizing the relationship between Tregs and surrounding cells, which might have implications for clinical outcomes and therapeutic strategies.

Our findings revealed that CD4^+^Tregs in TC as well as CD8^+^Tregs in IM and TC exhibited weaker interactions with surrounding tumor cells. Conversely, CD8^+^Tregs in IM and TC demonstrated stronger interactions with adjacent CD4^+^ T cells and CD8^+^ T cells. This phenomenon could potentially be attributed to the dynamic nature of the tumor microenvironment, as the immune milieu might be in an activated state of anti-tumor immunity during specimen collection. Subsequently, we conducted a survival analysis on the spatial characteristics of Tregs. The results revealed that strong interactions between Tregs and neighboring tumor cells in IM and TC were associated with adverse patient prognosis. Conversely, robust interactions between Tregs and adjacent CD4^+^T cells in TC and CD8^+^ T cells in IM and TC were correlated with favorable patient outcomes. These findings were consistent with prior research, where Tregs were known to foster an immunosuppressive niche that promoted tumor growth. Tregs could secrete inhibitory cytokines, such as interleukin-10 and transforming growth factor-β, attenuate the activity of nature killer (NK) cells and cytotoxic T cells and dampen the body’s anti-tumor immune surveillance [26–31]. Additionally, studies have shown that Tregs could exert direct cytolytic effects on NK cells and cytotoxic T cells through the action of granzyme B and perforin [31, 32]. Notably, strong interactions between CD4^+^Tregs in IM and TC and neighboring CD4^+^ T cells were associated with poor patient prognosis. This could potentially be attributed to the CD4^+^ T cells in this region being Th2 skewed, which migrates quickly and expresses elevated levels of anti-apoptotic signals, contributing to unfavorable patient outcomes [33–36]. These results underscored the significance of a precise analysis of short-range cell interactions in investigating the immunoregulatory role of Tregs.

Immunotherapies targeting NSCLC have garnered considerable attention in recent years, resulting in substantial advancements in patient outcomes. However, despite the considerable enthusiasm surrounding the success of these drugs, a significant proportion of patients fail to derive benefit from these treatments [37]. The expression of PD-L1 has limited predictive efficacy as an immunotherapy biomarker. Recent research on the interaction between PD-L1 and PD-1 has garnered significant attention. Sánchez-Magraner et al. reported that the interaction of the PD-1/PD-L1 but not the expression of PD-L1 is highly predictive of patient efficacy of immune treatment in NSCLC [38]. However, in our study, the interaction between PD-1 and PD-L1 in patients who did not receive immunotherapy appears to be unrelated to patient recurrence, suggesting that PD-L1/PD-1 interaction may only serve as a predictive marker of efficacy rather than prognosis. Therefore, future research should seek other more valuable biomarkers, aiming to guide patient stratification and treatment.

The application of immunotherapy has brought significant benefits to patients with NSCLC, and the impact of anti-PD-1/PD-L1 therapy on Tregs has also become a focal point of current research. Previous studies have demonstrated that the interaction between PD-1 and PD-L1 can promote the conversion of Th1 cells into inducible Tregs and selectively downregulate the expression of aminopeptidases, enhancing and sustaining FOXP3 expression on the surface of Tregs [39–41], which bolsters their immunosuppressive function. Our findings validated that the interaction between PD-1/PD-L1 is significantly stronger in the high FOXP3^+^ cells group, suggesting that this interaction fosters the proliferation and differentiation of Tregs, which is in line with prior research. Furthermore, in murine models, Yoshida et al. observed that ICI treatment blocking the PD-1/PD-L1 pathway effectively reduced the number of Tregs within tumors and increased tumor-infiltrating lymphocytes (TILs) [42]. Similarly, Toor et al. reported decreased FOXP3 expression on Treg cells’ surfaces in melanoma patients undergoing immunotherapy, consequently weakening the immunosuppressive function of Tregs [43]. However, Kamada et al. found that in patients receiving immunotherapy, tumor-infiltrating Tregs were significantly enhanced, leading to rapid tumor progression. The distinct regulatory effects of anti-PD-1/PD-L1 drugs on Tregs underscores the need for more in vivo and in vitro experiments to explore the impact of PD-1/PD-L1 interactions on Tregs [18]. This could potentially serve as a breakthrough in identifying efficacy markers for anti-PD-1/PD-L1 antibody therapy and target combination treatments contributing to precise therapeutic strategies for cancer patients.

Then, we created an SIS system that embraced diverse spatial immune features related to cell interactions, effectively portraying the intricate TME landscape and its influence on complex biological pathways. The SIS emerged as an autonomous and formidable prognostic marker for recurrence among patients with stages I-III NSCLC. It demonstrated notable precision in predicting recurrence outcomes across various TNM stages, aiding in the identification of individuals at heightened risk of early relapse. The SIS system put forth in the present investigation possessed a range of merits in contrast to alternative predictive frameworks. Firstly, the formulation of the SIS system integrated spatial immune features depicted through multi-color fluorescence staining which enabled a more faithful restoration of the TME and showcased commendable accuracy compared to features selected through solitary staining methods often employed in other studies. Secondly, due to the remarkable performance in clinical diagnosis and prognostication, diverse machine learning algorithms have gained extensive usage in predicting novel biomarkers and acquiring fresh insights. A combination of various machine learning offers an impartial approach to predicting patients’ clinical conditions [44–46].

Our study has several limitations. Firstly, our study lacked markers for other immune cell types within the TME, such as B cells, macrophages, and NK cells that could contribute to excavating more cell interaction network information to guide prediction and treatment. Second, we rarely detected the non-immune components in the TME to analyze these cells’ interaction with Tregs. Thirdly, our study was conducted in a single institute and external validation of the SIS system was lacking. Future investigations in multi-center settings involving diverse populations will be required to evaluate the robustness and credibility of our observations.

Future investigations ought to prioritize capturing the dynamic cellular composition and spatial architecture throughout the course of treatment, encompassing scenarios such as neoadjuvant and adjuvant immunotherapies. Moreover, the application of spatial transcriptomic and in vitro as well as in vivo experiments is imperative to meticulously unravel the intricate reconfiguration of the heterogeneous TME in response to diverse therapeutic interventions. In the context of the SIS system, forthcoming research endeavors should encompass a substantial sample size and treatment-related data to comprehensively assess its utility in predicting recurrence for patients afflicted with NSCLC.

## Conclusion

We established a framework for scrutinizing the cellular interaction networks within the TME using mIF images, which are capable of comprehending the biological events where spatial interactions among cells determine functionality. The spatial landscape that mixes distributed patterns among Tregs, tumor cells, CD4^+^T cells, and CD8^+^T cells were underscored to help in the stratification of patients who might be at risk of early recurrence.

## Supplementary Information

Below is the link to the electronic supplementary material.

Supplementary file1 (DOCX 582 KB)

Supplementary file2 (DOCX 26 KB)

## Data Availability

The datasets used and/or analyzed during the current study are available from the corresponding author upon reasonable request.

## Abbreviations

AUCs: Associated areas under the receiver-operating characteristic curve
CI: Confidence interval
FFPE: Formalin-fixed, paraffin-embedded
HR: Hazard ratio
IM: Invasive margin
LASSO: Least absolute shrinkage and selection operator
LUAD: Lung adenocarcinoma
LUSC: Lung squamous cell carcinoma
mIF: Multiplex immunofluorescence
mNND: Mean nearest neighbor distance
NK: Nature killer
NSCLC: Non-small cell lung cancer
PD-1: Programmed death-1
PD-L1: Programmed death ligand-1
RF: Random forest
RFS: Recurrence-free survival
ROC: Receiver-operating characteristic
ROI: Region of interest
SIS: Spatial immune signature
TC: Tumor center
Tcons: Conventional T cells
TMA: Tissue microarray
TME: Tumor microenvironment
Tregs: Regulatory T cells
XGBoost: Extreme gradient boosting
