# Supplementary material for: Spatial cell interplay networks of regulatory T cells predict recurrence in patients with operable non-small cell lung cancer
Source: Cancer Immunol Immunother. 2024 Aug 2;73(10):189. doi: 10.1007/s00262-024-03762-x (PMC11297009; doi:10.1007/s00262-024-03762-x)
Supplement: Supplementary file 1 — Supplementary file1 (DOCX 582 KB) [file 262_2024_3762_MOESM1_ESM.docx]

Supplementary file 1

**Treg-based spatial immune signature predict recurrence in patients with operable NSCLC**


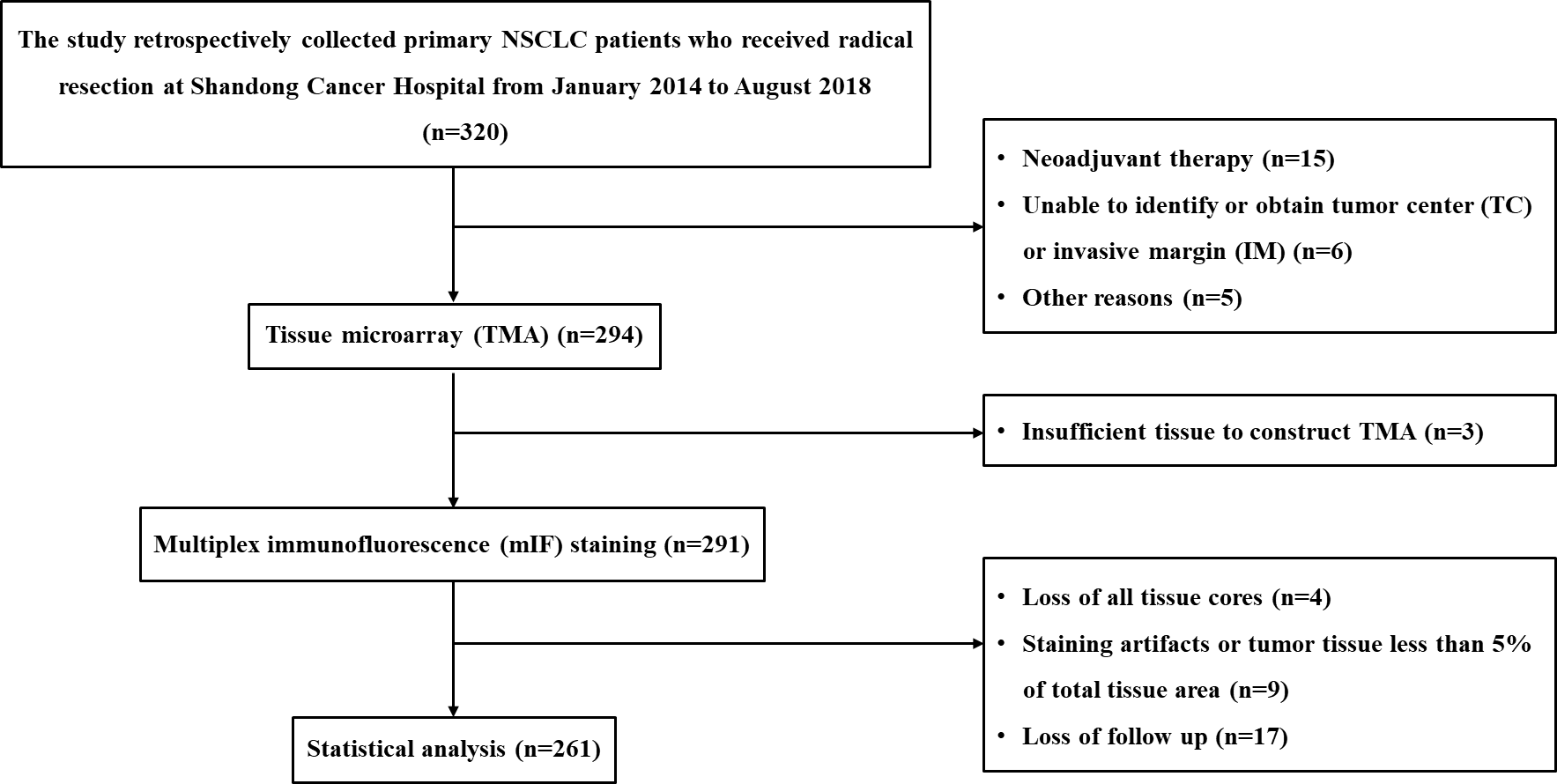


**Supplementary Figure S1.** Flowchart of the patient population in this study.


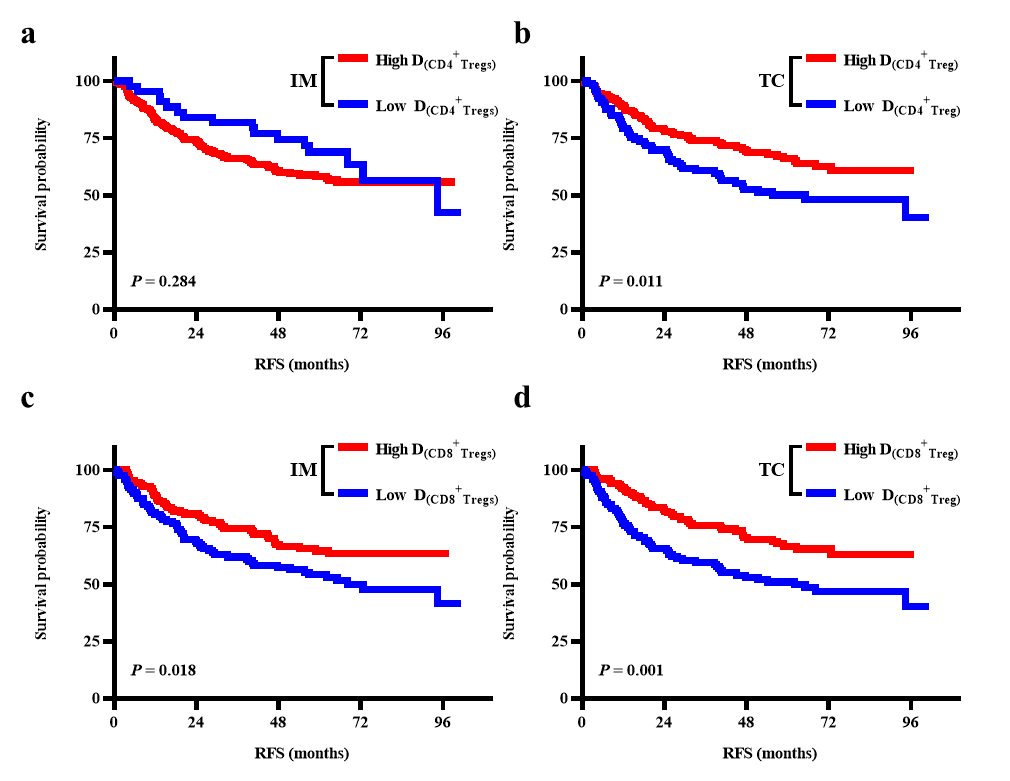


**Supplementary Figure S2.** The association of density of Tregs with RFS in NSCLC patients. (a-b) Kaplan-Meier curves of RFS based on the density of CD4^+^Tregs in IM and TC. (c-d) Kaplan-Meier curves of RFS based on the density of CD8^+^Tregs in IM and TC. Cumulative RFS were calculated by log-rank test.


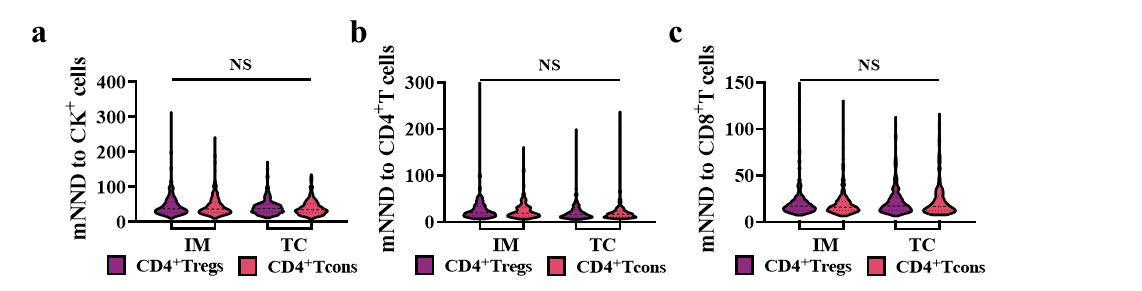


**Supplementary Figure S3.** Discrepancy of the mean nearest neighbor distance (mNND) from CD4^+^Tregs and CD4^+^Tcons to neighboring cells in IM and TC. (a) to CK^+^ cells. (b) to CD4^+^ T cells. (c) to CD8^+^ T cells. Significance (*P* value) was determined by Mann-Whitney U test. NS, no significance.


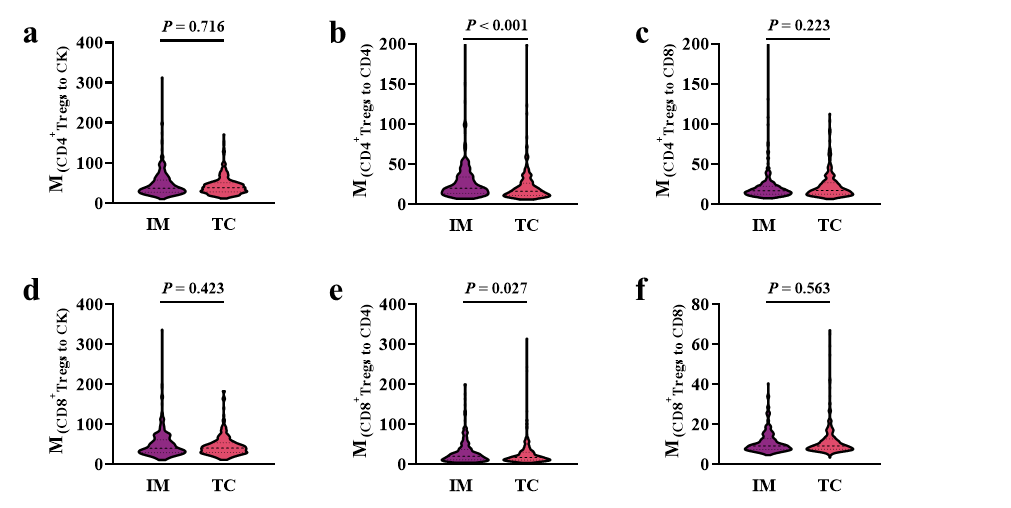


**Supplementary Figure S4.** Discrepancy of the mean nearest neighbor distance (mNND) from Tregs to neighboring cells between invasive margin and tumor center. **(a-c)** CD4^+^Tregs to neighboring cells. (d-f) CD8^+^Tregs to neighboring cells. Significance (*P* value) was determined by Mann-Whitney U test.


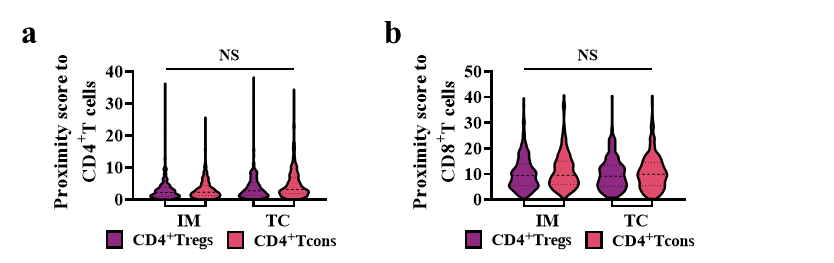


**Supplementary Figure S5.** Discrepancy of the mNND from CD4^+^Tregs and CD4^+^Tcons to neighboring cells in IM and TC. (a) to CD4^+^ T cells. (b) to CD8^+^ T cells. Significance (*P* value) was determined by Mann-Whitney U test. NS, no significance.


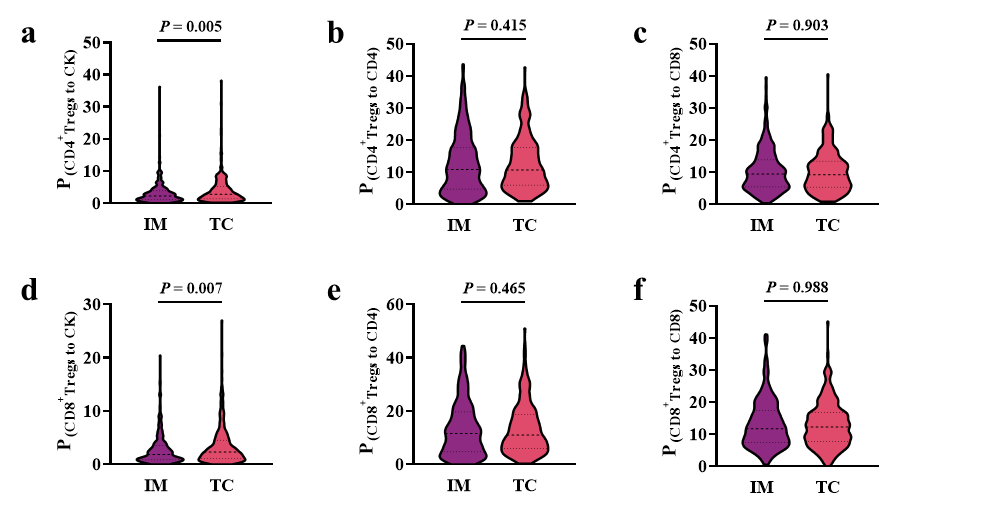


**Supplementary Figure S6.** Discrepancy of the proximity score from Tregs to neighboring cells between invasive margin and tumor center. **(a-c)** CD4^+^Tregs to neighboring cells. **(d-f)** CD8^+^Tregs to neighboring cells. Significance (*P* value) was determined by Mann-Whitney U test.

**
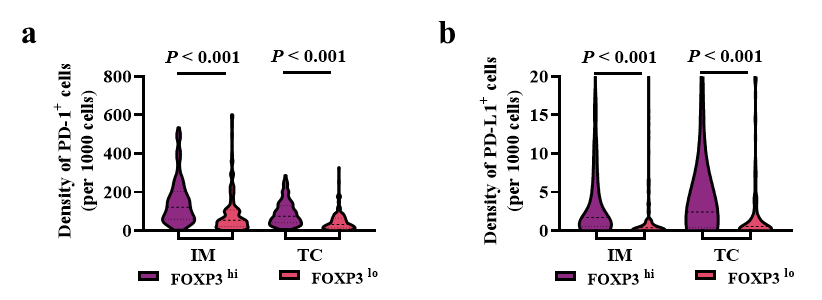
**

**Supplementary Figure S7.** Comparison of the density of PD-1^+^ cells or PD-L1^+^ cells between FOXP3 high expression group and FOXP3 low expression group. (a) PD-1^+^ cells. (b) PD-L1^+^ cells. Significance (*P* value) was determined by Mann-Whitney U test.

**
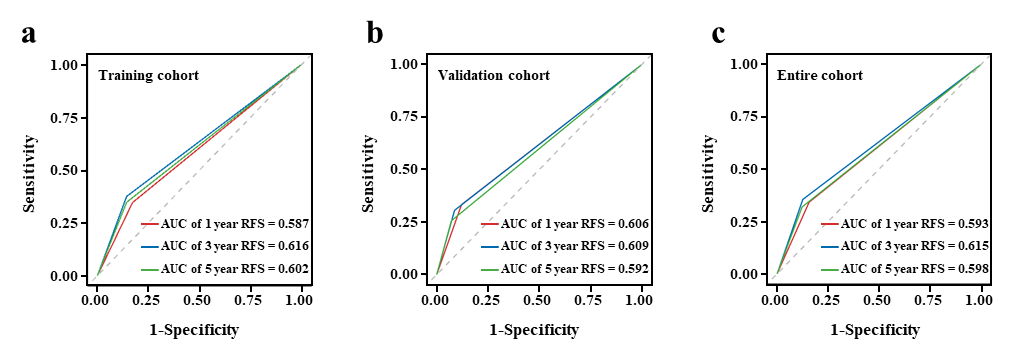
**

**Supplementary Figure S8.** Receiver and operating characteristic curves and area under curve values of TNM staging for prediction of recurrence risk at 1, 3 and 5 years. (a) in the training cohort. (b) in the validation cohort. (c) in the entire cohort.


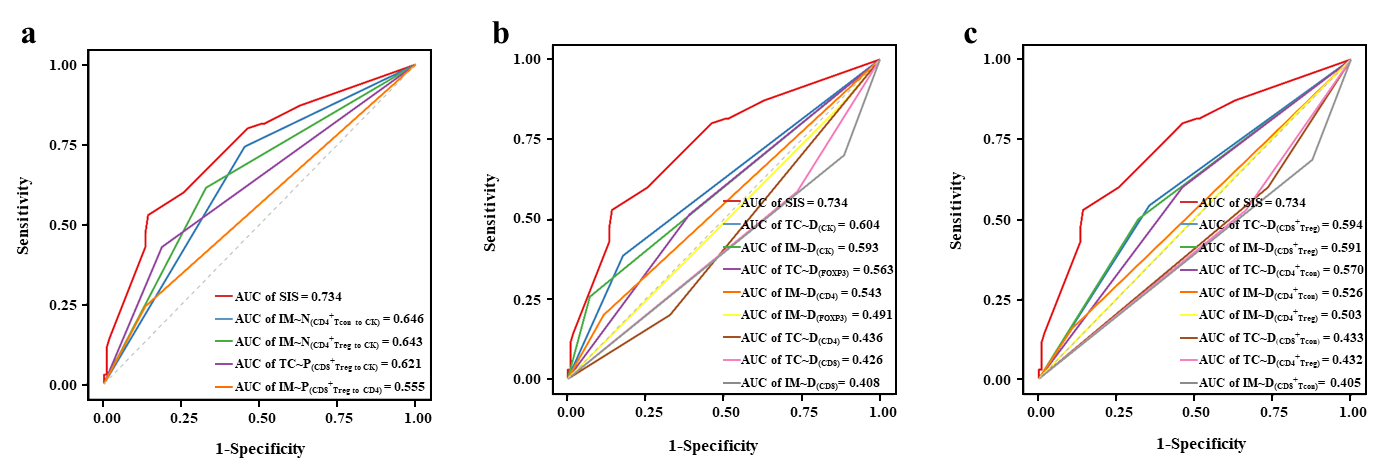


**Supplementary Figure S9.** Comparison of the predictive performance between SIS and single indicators using ROC curve analysis.

| **Supplementary Table S1. Information of primary antibodies used in the multiplex immunofluorescence test.** | | | | | | |
| --- | --- | --- | --- | --- | --- | --- |
| Reagent | Provider | | Identifier | | Concentration | |
| pan-CK | Zsbio | | ZM-0069 | | 1:200 | |
| CD4 | Zsbio | | ZM-0418 | | 1:1 | |
| CD8 | Abcam | | ab199016 | | 1:500 | |
| FOXP3 | Abcam | | ab20034 | | 1:100 | |
| PD-1 | Zsbio | | ZM-0381 | | 1:1 | |
| PD-L1 | Cell Signaling Technology | | #13684 | | 1:300 | |
| **Supplementary Table S2. Information of primary antibodies used in the multiplex immunofluorescence test.** | | | | | |  |
| Equipment | | Manufacturer | | Catalog number | |  |
| Constant Temperature Oven | | YAMATO | | DKN612C | |  |
| Ultrapure water meter | | Milli-Q | | Milli-Q IQ700 | |  |
| Fume hood | | BIOBOSE | | FH1500(A) | |  |
| Microwave oven | | Midea | | M1-L213B | |  |
| Centrifuge | | DLAB | | D1008E | |  |
| Vortex oscillator | | Kykin-Bell | | VORTEX-5 | |  |
| Shaker | | LABO | | HY-2A | |  |

| **Supplementary Table S3. Clinicopathological characteristics of 261 patients with samples subjected to multiplex staining.** | | | |
| --- | --- | --- | --- |
| **Parameter** | **Training Cohort**  **(N=183)** | **Validation Cohort**  **(N=78)** | ***P* value** |
| **Age, y** |  |  |  |
| ≤ 65 | 123 (67.2%) | 54 (69.2%) | 0.749 |
| > 65 | 60 (32.8%) | 24 (30.8%) |  |
| **Gender** |  |  |  |
| Male | 119 (65.0%) | 49 (62.8%) | 0.733 |
| Female | 64 (35.0%) | 29 (37.2%) |  |
| **Smoking index ^a^** |  |  |  |
| < 400 | 102 (55.7%) | 46 (59.0%) | 0.629 |
| ≥ 400 | 81 (44.3%) | 32 (41.0%) |  |
| **ECOG PS** |  |  |  |
| 0-1 | 50 (27.3%) | 22 (28.2%) | 0.884 |
| > 1 | 133 (72.7%) | 56 (71.8%) |  |
| **Histological type** |  |  |  |
| LUSC | 62 (33.9%) | 27 (34.6%) | 0.909 |
| LUAD | 121 (66.1%) | 51 (65.4%) |  |
| **T stage** |  |  |  |
| T1 | 63 (34.4%) | 24 (30.8%) | 0.542 |
| T2 | 101 (55.2%) | 48 (61.5%) |  |
| T3 | 11 (6.0%) | 2 (2.6%) |  |
| T4 | 8 (4.4%) | 4 (5.1%) |  |
| **N stage** |  |  |  |
| N0 | 129 (70.5%) | 54 (69.2%) | 0.221 |
| N1 | 27 (14.8%) | 17 (21.8%) |  |
| N2 | 27 (14.8%) | 7 (9.0%) |  |
| N3 | 0 (0%) | 0 (0%) |  |
| **AJCC stage** |  |  |  |
| I | 106 (57.9%) | 44 (56.4%) | 0.514 |
| II | 41 (22.4%) | 22 (28.2%) |  |
| III | 36 (19.7%) | 12 (15.4%) |  |
| **Adjuvant chemotherapy** |  |  |  |
| Yes | 116 (63.4%) | 49 (62.8%) | 0.931 |
| No | 67 (36.6%) | 29 (37.2%) |  |
| **Adjuvant radiotherapy** |  |  |  |
| Yes | 23 (12.6%) | 14 (17.9%) | 0.344 |
| No | 160 (87.4%) | 64 (82.1%) |  |

LUSC, lung squamous cell carcinoma; LUAD, lung adenocarcinoma.

a, Smoking index = number of cigarettes smoked per day × year(s).

b, Significance (*P* value) was determined by Chi-Squared test.

| **Supplementary Table S4. Univariate Cox regression demonstrated the prognostic effects of the density of Tregs.** | | |
| --- | --- | --- |
| **Variable** | **Univariate** | |
|  | **HR (95%CI)** | ***P*** |
| D_(CD4_^+^_Treg)_ in IM | 1.34 (0.79, 2.28) | 0.285 |
| D_(CD4_^+^_Treg)_ in TC | 0.61 (0.42, 0.90) | 0.011 |
| D_(CD8_^+^_Treg)_ in IM | 0.63 (0.43, 0.93) | 0.019 |
| D_(CD8_^+^_Treg)_ in TC | 0.54 (0.37, 0.78) | 0.001 |

| **Supplementary Table S5. Association of the mean nearest distance between Tregs and neighboring cells with clinicopathological factors.** | | | | | | |
| --- | --- | --- | --- | --- | --- | --- |
| **Proximity** | **Age**  **(≤ 65y vs. >65y)** | **Gender**  **(male vs. female)** | **Smoking index**  **(<400 vs. ≥400)** | **ECOG PS**  **(>1 vs. ≤1)** | **Histological subtype**  **(LUAD vs. LUSC)** | **TNM**  **(I vs. II vs. III)** |
| **N_(CD4_^+^_Treg to CK)_ in IM** | *P* = 0.417  (39.01 vs. 35.86) | *P* = 0.907  (37.11 vs. 37.84) | *P* = 0.107  (35.66 vs. 40.55) | ***P* = 0.015**  (39.16 vs. 33.07) | *P* = 0.869  (37.84 vs. 37.11) | ***P* < 0.001**  (42.84 vs. 36.37 vs. 28.96) |
| **N_(CD4_^+^_Treg to CK)_ in TC** | *P* = 0.264  (39.47 vs. 37.99) | *P* = 0.990  (39.26 vs. 37.88) | *P* = 0.799  (38.23 vs. 40.33) | *P* = 0.214  (39.55 vs. 37.19) | *P* = 0.165  (37.28 vs. 43.80) | ***P* < 0.001**  (42.79 vs. 36.71 vs. 28.89) |
| **N_(CD4_^+^_Treg to CD4)_ in IM** | *P* = 0.358  (19.69 vs. 21.96) | *P* = 0.254  (19.58 vs. 23.16) | ***P* = 0.009**  (22.76 vs. 19.08) | *P* = 0.925  (20.21 vs. 19.81) | ***P* = 0.002**  (22.22 vs. 18.02) | ***P* = 0.001**  (24.58 vs. 17.84 vs. 18.63) |
| **N_(CD4_^+^_Treg to CD4)_ in TC** | *P* = 0.263  (15.39 vs. 18.25) | ***P* < 0.001**  (18.33 vs. 12.84) | ***P* = 0.045**  (14.60 vs. 17.21) | *P* = 0.971  (15.95 vs. 17.10) | ***P* = 0.045**  (14.60 vs. 18.25) | *P* = 0.902  (16.30 vs. 15.31 vs. 17.18) |
| **N_(CD4_^+^_Treg to CD8)_ in IM** | *P* = 0.875  (17.12 vs. 16.06) | *P* = 0.596  (16.85 vs. 16.84) | ***P* = 0.033**  (17.95 vs. 15.54) | *P* = 0.051  (16.06 vs. 18.83) | *P* = 0.397  (17.24 vs. 16.34) | ***P* < 0.001**  (15.49 vs. 17.28 vs. 22.10) |
| **N_(CD4_^+^_Treg to CD8)_ in TC** | *P* = 0.846  (17.65 vs. 17.11) | ***P* = 0.028**  (18.76 vs. 16.47) | *P* = 0.433  (16.95 vs. 18.66) | *P* = 0.526  (17.11 vs. 18.45) | *P* = 0.252  (16.83 vs. 20.08) | ***P* < 0.001**  (15.32 vs. 19.50 vs. 27.06) |
| **N_(CD8_^+^_Treg to CK)_ in IM** | *P* = 0.924  (40.64 vs. 39.31) | *P* = 0.824  (41.10 vs. 39.16) | *P* = 0.096  (37.53 vs. 45.66) | ***P* = 0.018**  (42.68 vs. 35.32) | *P* = 0.683  (40.29 vs. 40.06) | ***P* = 0.001**  (46.28 vs. 38.29 vs. 32.63) |
| **N_(CD8_^+^_Treg to CK)_ in TC** | *P* = 0.364  (41.41 vs. 39.25) | *P* = 0.917  (41.23 vs. 40.18) | *P* = 0.850  (40.81 vs. 40.96) | *P* = 0.351  (41.23 vs. 38.86) | *P* = 0.297  (39.46 vs. 42.81) | ***P* < 0.001**  (44.72 vs. 38.52 vs. 33.34) |
| **N_(CD8_^+^_Treg to CD4)_ in IM** | *P* = 0.078  (19.92 vs. 25.86) | *P* = 0.294  (20.51 vs. 22.77) | ***P* = 0.038**  (23.87 vs. 19.11) | *P* = 0.269  (21.93 vs. 19.72) | ***P* < 0.001**  (24.43 vs. 16.99) | ***P* < 0.001**  (27.24 vs. 14.37 vs. 11.70) |
| **N_(CD8_^+^_Treg to CD4)_ in TC** | *P* = 0.314  (17.20 vs. 18.50) | ***P* < 0.001**  (18.86 vs. 13.80) | ***P* = 0.011**  (15.59 vs. 19.46) | *P* = 0.346  (17.91 vs. 16.82) | *P* = 0.089  (16.02 vs. 19.84) | ***P* = 0.012**  (18.76 vs. 15.95 vs. 13.97) |
| **N_(CD8_^+^_Treg to CD8)_ in IM** | *P* = 0.199  (9.03 vs. 9.80) | *P* = 0.188  (9.16 vs. 9.66) | *P* = 0.063  (9.65 vs. 9.09) | *P* = 0.630  (9.37 vs. 9.14) | *P* = 0.160  (9.46 vs. 9.09) | ***P* = 0.036**  (8.59 vs. 8.59 vs. 9.72) |
| **N_(CD8_^+^_Treg to CD8)_ in TC** | *P* = 0.740  (9.34 vs. 9.07) | *P* = 0.079  (9.33 vs. 9.02) | *P* = 0.397  (9.31 vs. 9.23) | *P* = 0.191  (9.74 vs. 8.65) | *P* = 0.803  (9.41 vs. 9.20) | *P* = 0.492  (10.23 vs. 8.88 vs. 9.31) |

| **Supplementary Table S6. Association of the proximity score between Tregs and neighboring cells with clinicopathological factors.** | | | | | | |
| --- | --- | --- | --- | --- | --- | --- |
| **Proximity** | **Age**  **(≤ 65y vs. >65y)** | **Gender**  **(male vs. female)** | **Smoking index**  **(<400 vs. ≥400)** | **ECOG PS**  **(>1 vs. ≤1)** | **Histological subtype**  **(LUAD vs. LUSC)** | **TNM**  **(I vs. II vs. III)** |
| **P_(CD4_^+^_Treg to CK)_ in IM** | *P* = 0.533  (2.21 vs. 2.55) | *P* = 0.123  (2.54 vs. 1.88) | *P* = 0.618  (2.25 vs. 2.25) | ***P* = 0.024**  (1.89 vs. 2.59) | *P* = 0.450  (2.20 vs. 2.55) | ***P* = 0.013**  (1.90 vs. 2.46 vs. 2.81) |
| **P_(CD4_^+^_Treg to CK)_ in TC** | *P* = 0.494  (2.80 vs. 2.75) | ***P* = 0.011**  (3.04 vs. 2.25) | *P* = 0.427  (2.74 vs. 3.04) | *P* = 0.462  (2.61 vs. 3.07) | *P* = 0.379  (2.58 vs. 3.10) | ***P* < 0.001**  (2.04 vs. 3.84 vs. 5.36) |
| **P_(CD4_^+^_Treg to CD4)_ in IM** | *P* = 0.331  (11.04 vs. 10.14) | *P* = 0.312  (11.31 vs. 10.02) | ***P* = 0.045**  (9.76 vs. 11.86) | *P* = 0.707  (11.00 vs. 10.02) | ***P* = 0.005**  (9.96 vs. 13.60) | ***P* = 0.040**  (9.47 vs.13.01vs. 11.89) |
| **P_(CD4_^+^_Treg to CD4)_ in TC** | *P* = 0.591  (10.80 vs. 10.36) | ***P* < 0.001**  (8.82 vs. 16.57) | ***P* = 0.001**  (12.47 vs. 9.11) | *P* = 0.534  (10.73 vs. 11.01) | ***P* = 0.038**  (10.85 vs. 10.09) | *P* = 0.253  (11.27 vs. 10.37 vs. 9.83) |
| **P_(CD4_^+^_Treg to CD8)_ in IM** | *P* = 0.211  (9.69 vs. 8.33) | *P* = 0.204  (9.87 vs. 8.24) | ***P* = 0.024**  (8.31 vs. 10.34) | ***P* = 0.023**  (9.55 vs. 7.78) | *P* = 0.308  (8.80 vs. 9.87) | ***P* = 0.003**  (9.73 vs. 10.42 vs. 6.24) |
| **P_(CD4_^+^_Treg to CD8)_ in TC** | *P* = 0.689  (9.33 vs. 8.74) | ***P* = 0.022**  (8.54 vs. 10.27) | *P* = 0.087  (9.57 vs. 8.81) | *P* = 0.373  (9.52 vs. 8.51) | *P* = 0.347  (9.53 vs. 8.13) | ***P* < 0.001**  (11.09 vs. 8.54 vs. 4.68) |
| **P_(CD8_^+^_Treg to CK)_ in IM** | *P* = 0.939  (1.94 vs. 1.70) | *P* = 0.197  (2.05 vs. 1.55) | *P* = 0.795  (1.88 vs. 1.80) | ***P* = 0.016**  (1.57 vs. 2.29) | *P* = 0.361  (1.67 vs. 2.06) | ***P* = 0.047**  (1.59 vs. 2.16 vs. 2.20) |
| **P_(CD8_^+^_Treg to CK)_ in TC** | *P* = 0.629  (2.34 vs. 2.16) | ***P* = 0.047**  (2.60 vs. 1.99) | *P* = 0.195  (2.15 vs. 2.40) | *P* = 0.607  (2.34 vs. 2.12) | *P* = 0.255  (2.24 vs. 2.60) | ***P* < 0.001**  (1.88 vs. 3.27 vs. 4.45) |
| **P_(CD8_^+^_Treg to CD4)_ in IM** | *P* = 0.172  (11.81 vs. 10.02) | *P* = 0.635  (11.73 vs. 10.42) | ***P* = 0.035**  (10.17 vs. 12.81) | *P* = 0.959  (11.00 vs. 12.14) | ***P* = 0.010**  (10.07 vs. 13.38) | ***P* = 0.001**  (8.02 vs. 13.98 vs. 13.49) |
| **P_(CD8_^+^_Treg to CD4)_ in TC** | *P* = 0.228  (12.08 vs. 9.75) | ***P* < 0.001**  (8.50 vs. 15.78) | ***P* < 0.001**  (13.53 vs. 8.81) | *P* = 0.517  (10.73 vs. 11.55) | ***P* = 0.039**  (12.63 vs. 9.47) | *P* = 0.652  (11.56 vs. 9.66 vs. 11.63) |
| **P_(CD8_^+^_Treg to CD8)_ in IM** | ***P* = 0.044**  (12.66 vs. 11.17) | *P* = 0.659  (12.15 vs. 11.11) | ***P* = 0.047**  (11.00 vs. 12.69) | *P* = 0.130  (12.18 vs. 11.40) | *P* = 0.217  (11.25 vs. 12.20) | *P* = 0.234  (11.91 vs. 12.98 vs. 10.88) |
| **P_(CD8_^+^_Treg to CD8)_ in TC** | *P* = 0.869  (12.39 vs. 10.57) | ***P* = 0.002**  (11.00 vs. 13.14) | ***P* = 0.015**  (12.48 vs. 11.36) | *P* = 0.923  (12.26 vs. 12.51) | *P* = 0.166  (12.39 vs. 10.57) | ***P* < 0.001**  (13.50 vs. 11.15 vs. 8.25) |

| **Supplementary Table S7. Multivariate Cox regression demonstrated the prognostic effects of the density of Tregs.** | | | | |
| --- | --- | --- | --- | --- |
| **Variable** | **Univariate** | | **Multivariate** | |
|  | **HR (95%CI)** | ***P*** | **HR (95%CI)** | ***P*** |
| N_(PD-L1 to PD-1)_ in IM | 0.69 (0.46, 1.03) | 0.072 | 0.72 (0.48, 1.08) | 0.113 |
| N_(PD-L1 to PD-1)_ in TC | 1.25 (0.85, 1.83) | 0.258 | 1.01 (0.70, 1.51) | 0.954 |
| P_(PD-L1 to PD-1)_ in IM | 0.71 (0.40, 1.25) | 0.232 | 0.71 (0.40, 1.24) | 0.222 |
| P_(PD-L1 to PD-1)_ in TC | 0.68 (0.46, 0.99) | 0.043 | 0.82 (0.55, 1.21) | 0.309 |

| **Supplementary Table S8. The indicators included in the model.** | | | |
| --- | --- | --- | --- |
| **Indicators in feature selection** | | | |
| **Density** | **Proximity score** | **Mean nearest neighboring distance** | |
| D_(FOXP3)_ in IM | P_(CD4_^+^_Treg to CK)_ in IM | N_(CD4_^+^_Treg to CK)_ in IM |  |
| D_(FOXP3)_ in TC | P_(CD4_^+^_Treg to CK)_ in TC | N_(CD4_^+^_Treg to CK)_ in TC |  |
| D_(CD4_^+^_Treg)_ in IM | P_(CD4_^+^_Treg to CD4)_ in IM | N_(CD4_^+^_Treg to CD4)_ in IM |  |
| D_(CD4_^+^_Treg)_ in TC | P_(CD4_^+^_Treg to CD4)_ in TC | N_(CD4_^+^_Treg to CD4)_ in TC |  |
| D_(CD8_^+^_Treg)_ in IM | P_(CD4_^+^_Treg to CD8)_ in IM | N_(CD4_^+^_Treg to CD8)_ in IM |  |
| D_(CD8_^+^_Treg)_ in TC | P_(CD4_^+^_Treg to CD8)_ in TC | N_(CD4_^+^_Treg to CD8)_ in TC |  |
| D_(CD4_^+^_Tcon)_ in IM | P_(CD4_^+^_Tcon to CK)_ in IM | N_(CD4_^+^_Tcon to CK)_ in IM |  |
| D_(CD4_^+^_Tcon)_ in TC | P_(CD4_^+^_Tcon to CK)_ in TC | N_(CD4_^+^_Tcon to CK)_ in TC |  |
| D_(CD8_^+^_Tcon)_ in IM | P_(CD4_^+^_Tcon to CD4)_ in IM | N_(CD4_^+^_Tcon to CD4)_ in IM |  |
| D_(CD8_^+^_Tcon)_ in TC | P_(CD4_^+^_Tcon to CD4)_ in TC | N_(CD4_^+^_Tcon to CD4)_ in TC |  |
| D_(CK)_ in IM | P_(CD4_^+^_Tcon to CD8)_ in IM | N_(CD4_^+^_Tcon to CD8)_ in IM |  |
| D_(CK)_ in TC | P_(CD4_^+^_Tcon to CD8)_ in TC | N_(CD4_^+^_Tcon to CD8)_ in TC |  |
| D_(CD4)_ in IM | P_(CD8_^+^_Treg to CK)_ in IM | N_(CD8_^+^_Treg to CK)_ in IM |  |
| D_(CD4)_ in TC | P_(CD8_^+^_Treg to CK)_ in TC | N_(CD8_^+^_Treg to CK)_ in TC |  |
| D_(CD8)_ in IM | P_(CD8_^+^_Treg to CD4)_ in IM | N_(CD8_^+^_Treg to CD4)_ in IM |  |
| D_(CD8)_ in TC | P_(CD8_^+^_Treg to CD4)_ in TC | N_(CD8_^+^_Treg to CD4)_ in TC |  |
| D_(PD-L1)_ | P_(CD8_^+^_Treg to CD8)_ in IM | N_(CD8_^+^_Treg to CD8)_ in IM |  |
| D_(PD-1)_ | P_(CD8_^+^_Treg to CD8)_ in TC | N_(CD8_^+^_Treg to CD8)_ in TC |  |
| - | P_(CD8_^+^_Tcon to CK)_ in IM | N_(CD8_^+^_Tcon to CK)_ in IM |  |
| - | P_(CD8_^+^_Tcon to CK)_ in TC | N_(CD8_^+^_Tcon to CK)_ in TC |  |
| - | P_(CD8_^+^_Tcon to CD4)_ in IM | N_(CD8_^+^_Tcon to CD4)_ in IM |  |
| - | P_(CD8_^+^_Tcon to CD4)_ in TC | N_(CD8_^+^_Tcon to CD4)_ in TC |  |
| - | P_(CD8_^+^_Tcon to CD8)_ in IM | N_(CD8_^+^_Tcon to CD8)_ in IM |  |
| - | P_(CD8_^+^_Tcon to CD8)_ in TC | N_(CD8_^+^_Tcon to CD8)_ in TC |  |
| - | P_(PD-L1 to PD-1)_ in IM | N_(PD-L1 to PD-1)_ in IM |  |
| - | P_(PD-L1 to PD-1)_ in TC | N_(PD-L1 to PD-1)_ in TC |  |
