# Supplementary material for: Spatial cell interplay networks of regulatory T cells predict recurrence in patients with operable non-small cell lung cancer
Source: Cancer Immunol Immunother. 2024 Aug 2;73(10):189. doi: 10.1007/s00262-024-03762-x (PMC11297009; doi:10.1007/s00262-024-03762-x)
Supplement: Supplementary file 2 — Supplementary file2 (DOCX 26 KB) [file 262_2024_3762_MOESM2_ESM.docx]

Supplementary file 2

**Treg-based spatial immune signature predict recurrence in patients with operable NSCLC**

**The detailed information of automated multispectral image processing was illustrated as follows:**

Multispectral images were processed using the Inform software 2.4.8 (PerkinElmer, USA).

Initially, the mixed multispectral images underwent spectral unmixing to generate single-spectral images. This process utilized a fluorescence spectral library derived from singly stained samples for each fluorophore. To address nonspecific autofluorescence, regions lacking staining within the images were identified and subtracted before tissue segmentation.

Secondly, the distinction between the tumor compartment and stroma compartment was visually determined by evaluating the signal of the epithelial cell marker (pan-CK) in a minimum of 10 images. This process was essential to train a tissue classifier, which was subsequently applied to segment all images. The accuracy threshold for the trained tissue classifier was set at exceeding 95% (achieving 98.85% accuracy in our customized project).

Thirdly, considering the heterogeneous mixtures of cell types with distinct nuclear and cell morphologies present in the images, we opted for an adaptive cell segmentation approach. This involved integrating nuclear signals (DAPI, Foxp3), membrane signals (CD8, CD4), and cytoplasmic signal (CK) to achieve comprehensive segmentation.

Lastly, like tissue segmentation, we trained a single-marker phenotype classifier by visually identifying a minimum of 30 positive cells and 30 negative cells for each marker. Subsequently, all images underwent processing using the trained phenotype classifier, with an accuracy requirement set to exceed 95% (surpassing 99.50% in our customized project for each marker).

Following the aforementioned procedures, single-marker phenotypes and single-cell two-dimensional coordinates were generated. All hematoxylin-eosin and mIF images underwent independent review by at least two investigators, including an experienced pathologist. Quantitative and spatial parameters of cells were utilized to elucidate the features of intratumoral cell diversity. To quantify cell types defined by multiple-marker phenotypes and determine nucleus-to-nucleus Euclidean distances between different cell types, we employed a bioinformatics tool (phenoptr 0.3.2; https://github.com/akoyabio/phenoptr).
